# Case of Peritonæal Inflammation

**Published:** 1858-07

**Authors:** W. C. Haines

**Affiliations:** Hicks Mills, Ill.


ARTICLE VI.

### CASE OF PERITONÆAL INFLAMMATION.

BY W. C. HAINES, M. D., OF HICKS MILLS, ILL.

Eds. Chicago Medical Journal,—In the April number of your journal, over the signature of "B.," I found some excellent and truthful remarks in relation to the duties which the members of the medical profession owe to each other, in communicating their experience to the medical journals of the day. The subject certainly is a very important one, and which should meet with a hearty response from the profession generally, and every member should feel himself individually called upon to aid and cooperate in effecting so desirable an object. There is accumulating all over the country a vast amount of valuable and important information, which, if it could be brought to light, might be easily made available, through the medium of a medical journal, for some useful purpose. If every reader of the Chicago Journal would send to its editors a condensed report of the most important and interesting cases occurring in his practice during the year, or previous years, as well as all items of important information which he may be in possession of, and indeed anything that could in any way be of service to the profession, it would not be long before there would accumulate a mass of material which no estimate could be put upon its value. It is to this end that a medical journal proves such a valuable auxiliary. "B.," in his remarks, calls on the profession to write! write! I

have done so, and you can put it into type or into the fire as you think best. A report of one case must, however, suffice for the present time, yet more anon.

On the morning of the 16th May, 1856, a messenger came for me to go and see Mrs. M., who was dangerously sick and not expected to live. Not being at home, I did not see her till near night; on my arrival, I found her, to all appearance, in a sinking condition, not able to converse above a whisper; pulse very feeble; skin hot and dry, with a dry, red tongue; abdomen much enlarged, hot and tender. Had been sick a number of days. A physician had been in attendance, treating her for an ovarian tumor. From the history I obtained from my patient's friends, I had my doubts as to the correctness of his diagnosis. I gave my opinion that her disease was peritonæal inflammation, and prescribed accordingly, giving my patient full doses of opium every three hours, and frequent application of cold water to abdomen. No other remedies were used. She made a quick recovery, being quite free from all ovarian disease. Since that time my patient's abdomen increased in size somewhat, yet time and nature were competent to effect a cure. Among the various remedies recommended for the treatment of peritonæal inflammation, there are none that has established a confidence in my mind equal to opium given in full doses.

